# Hybrid Integration Approach of Entropy with Logistic Regression and Support Vector Machine for Landslide Susceptibility Modeling

**DOI:** 10.3390/e20110884

**Published:** 2018-11-17

**Authors:** Tingyu Zhang, Ling Han, Wei Chen, Himan Shahabi

**Affiliations:** 1 School of Earth Science and Resources, Chang’an University, Key Laboratory of Degraded and Unutilized Land Remediation Engineering, Ministry of Land and Resources, Shaanxi Provincial Key Laboratory of Land Rehabilitation, Xi’an 710064, Shaanxi, China; 2 College of Geology & Environment, Xi’an University of Science and Technology, Xi’an 710054, Shaanxi, China; 3 Department of Geomorphology, Faculty of Natural Resources, University of Kurdistan, Sanandaj 66177-15175, Iran

**Keywords:** landslides, hybrid model, statistical method, machine learning, loess area

## Abstract

The main purpose of the present study is to apply three classification models, namely, the index of entropy (IOE) model, the logistic regression (LR) model, and the support vector machine (SVM) model by radial basis function (RBF), to produce landslide susceptibility maps for the Fugu County of Shaanxi Province, China. Firstly, landslide locations were extracted from field investigation and aerial photographs, and a total of 194 landslide polygons were transformed into points to produce a landslide inventory map. Secondly, the landslide points were randomly split into two groups (70/30) for training and validation purposes, respectively. Then, 10 landslide explanatory variables, such as slope aspect, slope angle, altitude, lithology, mean annual precipitation, distance to roads, distance to rivers, distance to faults, land use, and normalized difference vegetation index (NDVI), were selected and the potential multicollinearity problems between these factors were detected by the Pearson Correlation Coefficient (PCC), the variance inflation factor (VIF), and tolerance (TOL). Subsequently, the landslide susceptibility maps for the study region were obtained using the IOE model, the LR–IOE, and the SVM–IOE model. Finally, the performance of these three models was verified and compared using the receiver operating characteristics (ROC) curve. The success rate results showed that the LR–IOE model has the highest accuracy (90.11%), followed by the IOE model (87.43%) and the SVM–IOE model (86.53%). Similarly, the AUC values also showed that the prediction accuracy expresses a similar result, with the LR–IOE model having the highest accuracy (81.84%), followed by the IOE model (76.86%) and the SVM–IOE model (76.61%). Thus, the landslide susceptibility map (LSM) for the study region can provide an effective reference for the Fugu County government to properly address land planning and mitigate landslide risk.

## 1. Introduction

Landslides often occur in mountainous and hilly areas and are one of the most dangerous geological disasters [1]. Landslides can cause huge economic losses and a large number of casualties. According to statistics, almost 1000 people and 4 billion dollars are lost annually in the world [2], and this figure still keeps growing. China is also a region where landslides frequently occur; it has been reported that 7122 geological disasters occurred in 2017, causing 327 deaths, 173 injured, 25 missing, and a loss of 3.54 billion CNY [3]. In addition, in northwestern China, landslides pose a greater threat to resident security and transportation, because of the harsh environment and population concentration. However, enormous manpower and material resources may be required to control and renovate every landslide. Therefore, predicting landslide occurrence is both valuable and important.

As the first step to predicting landslide occurrences, a landslide susceptibility analysis aims to recognize hazardous and high-risk regions, and a preference for the negative effects of landslides [4]. The landslide susceptibility map (LSM) is the final result of the landslide susceptibility analysis. However, the traditional methods for landslide susceptibility mapping based on filed investigation and manual analysis are time-consuming and expensive, and the result is imprecise [5,6]. In recent years, geographical information systems (GIS) have been vigorously developed, which make the preparation of the landslide susceptibility map more convenient, which has great advantages [7]. Meanwhile, there has been a lot of research on the combination of geographical information systems, and statistical and nonstatistical methods to evaluate landslide susceptibility—in terms of the binary statistical method, for example, the frequency ratio (FR) model [8,9,10,11,12,13], the certainty factor (CF) model [14,15,16,17], the statistical index (SI) [18,19], the weights of evidence (WOE) [20,21,22], and the index of entropy (IOE) model [23,24]. The factor internal coefficient of certainty or weight of evidence is decided by landslide data, but the selection of factors would be influenced by humans. As a multivariate statistical method, the logistic regression (LR) model is extensively applied by many researchers [25,26,27,28,29,30].

Due to the limitation of statistical models, some machine learning algorithms that can avoid the influence from humans were also introduced and applied for landslide susceptibility analysis, such as artificial neural networks (ANN) [31,32,33], neuro-fuzzy [34,35,36,37], fuzzy logic [38,39], decision trees [40,41,42], kernel logistic regression (KLR) [43,44], and support vector machines (SVM) [45,46,47].

Statistical models and machine learning algorithms have their own advantages and disadvantages [48,49]. The internal parameters of the explanatory variables in binary statistical models are determined by landslide data, which can avoid the interference of human factors and be more objective. However, the selection of explanatory variables will receive interference from humans. By contrast, multivariable statistical models and machine learning methods can avoid the problem of factor dependence, but they are less widespread and limited to few cases of study for their intensive computation [50,51]. In recent years, many hybrid models have been used in the literature, such as the fuzzy weight of evidence method [17], adaptive network-based fuzzy inference system (ANFIS) based on frequency ratio (FR–ANFIS) model [52], wavelet packet–statistical (WP–SM) models [53], and integration of support vector machines and the multiboost [54]. According to plenty of research, the hybrid model generally performed better than the original models, so trying to mix different models and apply them to different regions is significant. Therefore, this research assembled the IOE model with the LR and SVM models to form two hybrid models (LR–IOE and SVM–IOE) for landslide susceptibility mapping in the Fugu County of Shaanxi Province, China.

## 2. Study Area

**Figure 1:**
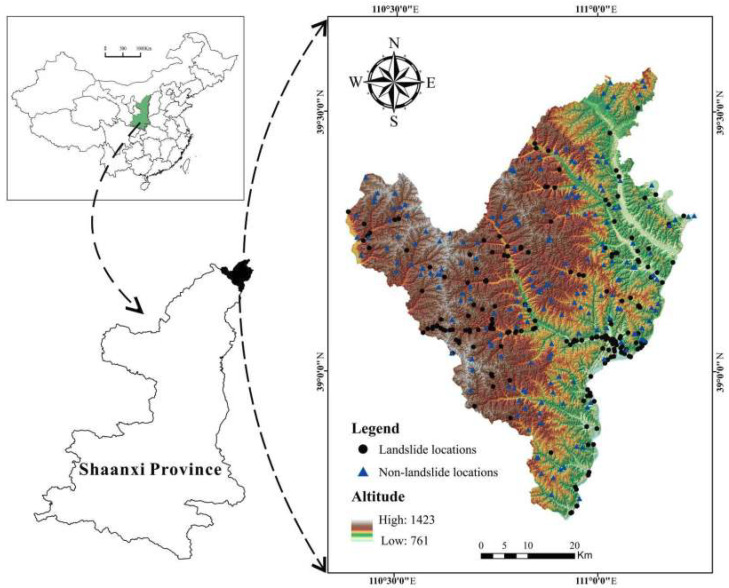
Landslide inventory map and the location of study area.

The Fugu County, whose geographic coordinates are 110°25′ to 111°15′ east longitude and 38°42′ to 39°33′ north latitude, covers an area of 3229 Km^2^ (Figure 1). The elevation in the study area is between 761 and 1423 m above sea level, and increases from east to west. The temperate zone with an arid continental monsoon climate is the main climate type in the study region, and the maximum and minimum temperatures in history are 38.9 °C and −24 °C, while the average annual temperature is 9.1 °C. The average annual rainfall is 428.6 mm, and the geographical distribution of rainfall shows a gradual increase from northwest to southwest. Meanwhile, most of the precipitation is concentrated from July to September, accounting for 69% of the annual rainfall. There are 62 rivers with drainage areas above 1 × 10^7^ m^2^ in the study region, and the average annual runoff is 5.911 × 10^9^ m^3^.

The overall topography of the study area is high in the northwest and low in the southwest. The main landform types can be divided into four types as follow: Loess girder landform, loess gully landform, canyon hilly landform, and valley terraces. The dip direction of rock formation is roughly southwest–northwest, with a dip angle of approximately 5–8 degrees except for a few areas, which are about 20 degrees. The Carboniferous–Permian strata in the east and the Jurassic strata in the northwest are coal-bearing strata, and the lithology in the study area is shown in Table 1.

Due to the rich coal resources in the study area, the mining industry is developed and the population is concentrated, which caused serious damage to the environment. At the same time, it has also formed massive landslides.

## 3. Data Used

### 3.1. Landslide Inventory Map

A landslide inventory map is the first step in a landslide susceptibility analysis and includes historical and newly discovered landslides and their relational information [43], such as the location, the date of occurrence, the extent of landslide phenomena in a region, and the types of mass movements that have left discernable traces [55]. In order to obtain a practical and accurate landslide inventory map, data collection and an adequate field survey were significantly in the current study. A digital elevation model (DEM) of the study region with 30 m resolution was obtained from ASTER GDEM, downloaded from Geospatial Data Cloud [56]. The geological map and mean annual precipitation data were provided by the government of Fugu County. Based on field investigations, a total of 194 landslides polygons, including 162 slides, 29 falls, and 3 debris flows, were drawn according to the depletion zone, and these landslides were triggered by rainfall and excavation. In the study area, the smallest and largest sizes of these landslides were about 39 m^2^ and 13.5 × 10^4^ m^2^, respectively. Because only 12% of landslides are over 10,000 m^2^ in size, landslide polygons were transformed into points using the centroid method and then the landslide inventory map (Figure 1) was obtained in the present study [57,58].

To avoid the overfitting problems in modeling, a total of 194 nonlandslide points were randomly generated and mapped on the landslide inventory map. All of these landslide and nonlandslide points were randomly divided into two groups; namely, the training dataset, including 272 (70%) points, was used to train the models, and the validating dataset, including 116 (30%) points, was used for validation propose.

### 3.2. Landslide Explanatory Variables

In order to produce the landslide susceptibility map, 10 landslide explanatory variables, namely slope aspect, altitude, slope angle, lithology, mean annual precipitation, distance to roads, distance to rivers, distance to faults, land use, and normalized difference vegetation index (NDVI), were selected to produce data layers representing themselves with a resolution of 30 × 30 m. Slope aspect, altitude, and slope angle maps were extracted from DEM data using ArcGIS software. Land use and NDVI were extracted from GF-2 satellite images gathered from the China Center for Resources Satellite Data and Application. Lithology, distance to roads, mean annual precipitation, distance to rivers, and distance to faults maps were extracted based on existing data.

**Figure 2:**
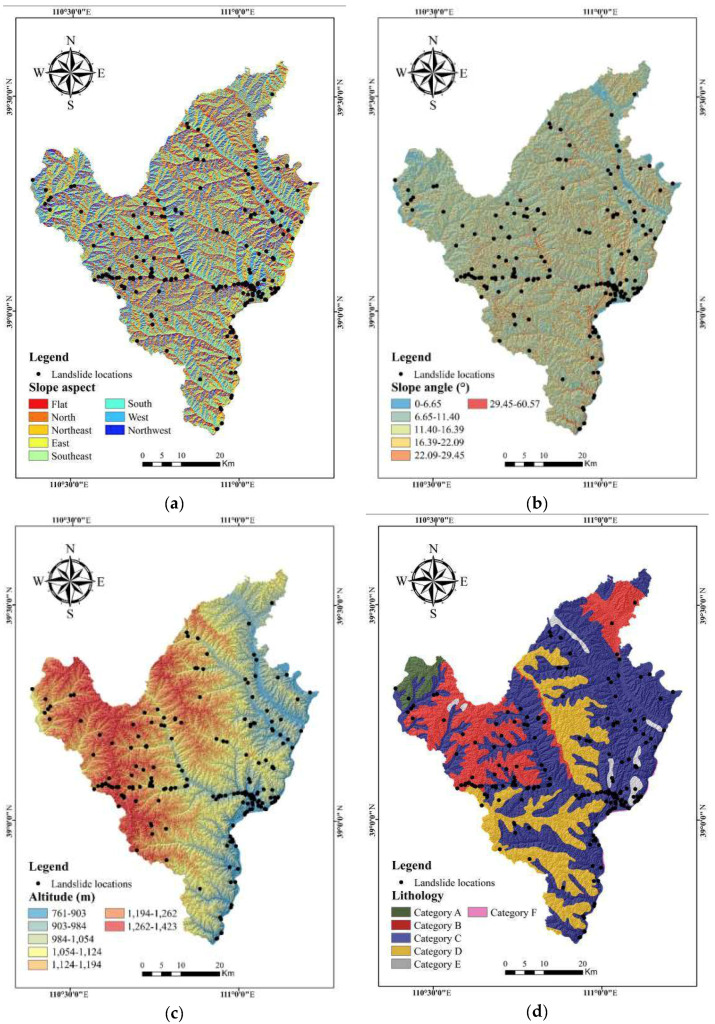

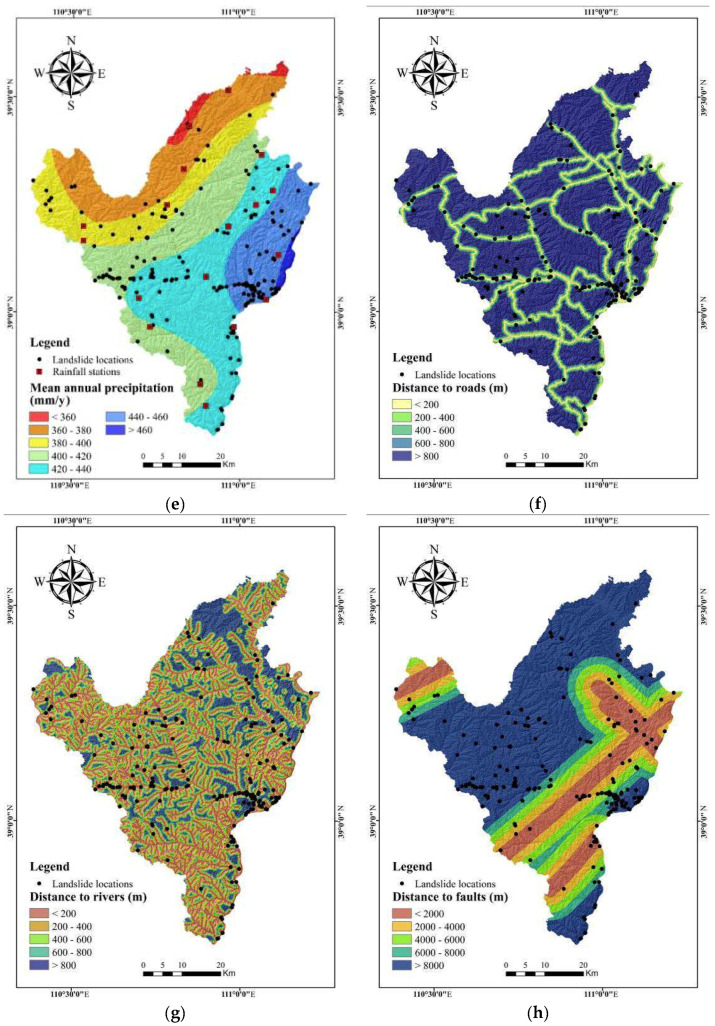

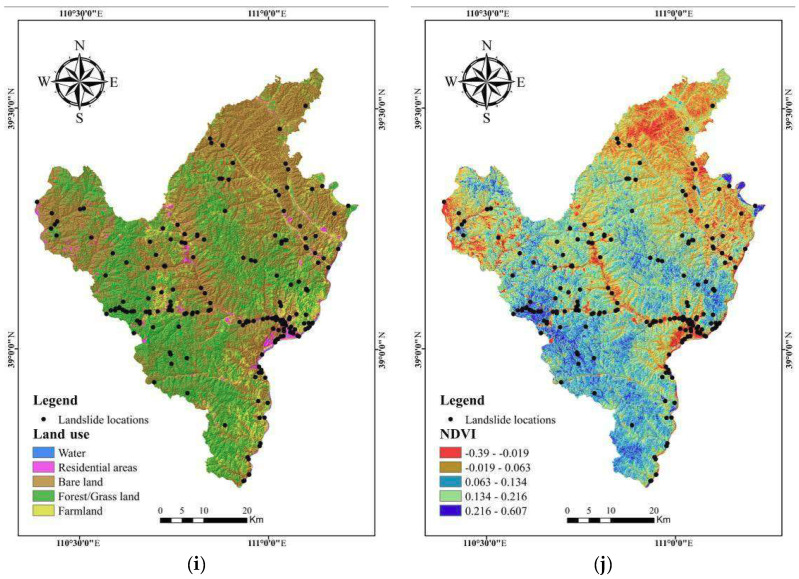
Landslide explanatory variable maps involving: (**a**) Slope aspect; (**b**) slope angle; (**c**) altitude; (**d**) lithology; (**e**) mean annual precipitation; (**f**) distance to roads; (**g**) distance to rivers; (**h**) distance to faults; (**i**) land use; (**j**) normalized difference vegetation index (NDVI).

The slope aspect, which is considered to be a prerequisite condition, was frequently adopted by many works in the literature to produce a landslide susceptibility map [30]. The slope aspect was reclassified into nine groups, based on the equal interval method, as follows: Northwest, west, southwest, south, southeast, east, northeast, north, flat, respectively (Figure 2a).

As it is considered to be another critical factor, the slope angle was widely used by a lot of relevant research [59]. In the current research, the slope angle was divided into the following six categories, based on the Jenks natural break method, as follows: 0°–6.65°, 6.65°–11.40°, 11.40°–16.39°, 16.39°–22.09°, 22.09°–29.45°, 29.45°–60.57° (Figure 2b).

Altitude is also considered a significant factor for landslide susceptibility mapping [1]. Thus, based on the Jenks natural break method, elevation values were classified into the following seven ranges: 761–903 m, 903–984 m, 984–1054 m, 1054–1124 m, 1124–1194 m, 1194–1262 m, and 1262–1423 m (Figure 2c).

The difference of lithology is the basis of landslide formation conditions [60]. According to field investigations and the existing geological data and maps, lithological units were divided into six categories (Table 1) and the lithology map was produced (Figure 2d).

Previous research has indicated that there is a strong correlation between mean annual precipitation and landslide occurrences [61,62,63]. According to the existing and local observation data, mean annual precipitation is divided into seven classes based on equal interval method as follows: <360 mm/y, 360–380 mm/y, 380–400 mm/y, 400–420 mm/y, 420–440 mm/y, 440–460 mm/y, and >460 mm/y (Figure 2e).

Distance to roads is used as an important landslide explanatory variable to prepare the distance to roads map [64]. In this study, the values of distance to roads were reclassified into five ranges based on equal interval method as follows: <200 m, 200–400 m, 400–600 m, 600–800 m, and >800 m (Figure 2f).

River erosion of slope is considered to be a significant explanatory variable inducing landslides; thus, distance to rivers is employed to be a quantitative index of river erosion [25]. In this study, with 200 m as the interval, the values of distance to rivers were reclassified into five ranges based on equal interval method as follows: <200 m, 200–400 m, 400–600 m, 600–800 m, and >800 m (Figure 2g).

Fault movement is not only the requirement for individual landslide occurrences, but also a controlling factor for regional landslide occurrences [12]. A mass of field surveys indicated that the more fault movement occurred acutely, the more landslides were triggered. In the current research, with 2000 m as the interval, the values of distance to faults were reclassified into five ranges based on equal interval method as follows: <2000 m, 2000–4000 m, 4000–6000 m, 6000–8000 m, and >8000 m (Figure 2h).

Land use in different regions will be different. The use of these land may lead to an asymmetrical distribution of landslides [65]. Thus, land use was also employed to be an explanatory variable in the study region, which was generally divided into five categories as follows: Water, residential areas, bare land, forest/grassland, and farmland (Figure 2i).

NDVI reflects the surface condition and provides a quantitative estimate of vegetation growth and biomass. This is depending on the biomass, the position within the hillslope profile, the root-zone depth and possibility to crack rocks and to prevent or ease water infiltration [66,67]. Therefore, NDVI is also considered to be a pivotal explanatory variable. The computational formula of NDVI is defined as follows:

(1) $$\mathrm{NDVI}=\frac{\mathrm{NIR}-R}{\mathrm{NIR}+R},$$

where R stands for the red part of electromagnetic spectrum, while NIR represents the infrared part of electromagnetic spectrum. Using the Jenks natural break method, the NDVI values were reclassified into five categories as follows: −0.39 to −0.019, −0.019 to 0.063, 0.063–0.134, 0.134–0.216, and 0.216–0.607 (Figure 2j).

## 4. Methodologies

### 4.1. Multicollinearity Diagnosis

In the study region, not all explanatory variables have a positive impact on the classification results. Multicollinearity problems may exist between explanatory variables, which may lead to an overfit in modeling. Thus, the Pearson correlation coefficient (PCC), the variance inflation factor (VIF), and tolerance (TOL) were introduced to detect the potential multicollinearity problems [68].

The essence of PCC is a statistical linear correlation coefficient, and its analysis is usually used to measure the linear relationship between distance variables. For two sets of samples *X_i_* (*i* = 1, 2, 3, ..., *n*) and *Y_j_* (*j* = 1, 2, 3, ..., *n*), the PCC between them can be expressed as:

(2) $$\mathrm{PCC}=\frac{\sum_{i=1}^{n}(x_{i}-\bar{x})\sum_{j=1}^{n}\left(y_{j}-\bar{y}\right)}{\sqrt{\sum_{i=1}^{n}{\left(x_{i}-\bar{x}\right)}^{2}\sum_{j=1}^{n}{\left(y_{i}-\bar{y}\right)}^{2}}},$$

where *x_i_* and *y_j_* are variable values for *X_i_* and *Y_j_*. $\bar{x}$ and $\bar{y}$ are the average of *X_i_* and *Y_j_*, respectively. In general, the greater the absolute value of PCC is, the higher the risk of multicollinearity between the landslide explanatory variables [69], and a PCC of >0.7 indicates a multicollinearity problem [70].

The VIF and TOL are two important indexes for a multicollinearity diagnosis. VIF refers to the ratio of the variance when there is multicollinearity between the conditioning factors and the variance when there is no multicollinearity, and the tolerance is the reciprocal of VIF [71]. In general, the larger the VIF values and the smaller the tolerances values are, the stronger the multicollinearity between the conditioning factors. In this study, the explanatory variables with VIF >2 or TOL <0.4 should be abandoned [72].

### 4.2. Index of Entropy (IOE) Method

The first classification model applied in the present study is the index of entropy (IOE) model, which is a bivariate statistic model; the IOE is also used to be the input data to build the hybrid models in the subsequent modeling. The entropy means the degree of unsteadiness and indeterminacy of a system, and also indicates that elements in a natural environment are the most related development for mass movement [23]. In addition, the entropy represents the degree of different explanatory variables that affect the development of landslides in a landslide susceptibility analysis. The weight values (*W_j_*) of each landslide explanatory variable are determined by the following equations [73]:

(3) $$FR_{ij}=\frac{y_{ij}}{x_{ij}},$$

(4) $$S_{ij}=\frac{FR_{ij}}{\sum_{j=1}^{N_{j}}FR_{ij}},$$

(5) $$M_{j}=-\sum_{i=1}^{N_{j}}S_{ij}\log_{2}S_{ij},j=1,2,3,...,n,$$

(6) $$M_{j\max}=\log_{2}N_{j},$$

(7) $$I_{j}=\frac{M_{j\max}-M_{j}}{M_{j\max}},$$

(8) $$W_{j}=I_{j}\times FR_{ij},$$

where *FR_ij_* is the frequency ratio value; *x* and *y* represent the percentage of domain and percentage of landslides, respectively; *S_ij_* stands for the probability density; entropy values are represented by *M_j_* and *M_jmax_*; *N_j_* means the number of categories or ranges of each explanatory variables; and *I_j_* is the information parameters.

Then, the final weight values are calculated by SPSS software. Because these three explanatory variables (aspect, lithology, and land use) are generated from vector graphics with no attribute values, the *FR* values of aspect, lithology, and land use were used as input data for the computation of *W_j_*. Finally, the landslide susceptibility map for the IOE model is produced using the following equation:

(9) $${\mathrm{LSI}}_{\mathrm{IOE}}=\sum_{j=1}^{n}\frac{e}{f_{j}}\times C\times W_{j},$$

where LSI_IOE_ stands for the sum of all the categories; *j* represents the number of explanatory variable maps; *e* means the number of classes within explanatory variable maps with the greatest number of groups; *f_j_* is the number of classes within particular explanatory variable maps; and *C* indicates the value of the categories after secondary classification [74].

### 4.3. Integration of Logistic Regression and Index of Entropy Model

The logistic regression (LR) model is employed to integrate with the IOE to build a new hybrid model, namely, the LR–IOE model in this study. Logistic regression is a commonly used statistical analysis method for regression analysis of binary classification dependent variables. The superiority of the LR model is that independent variables can be discrete or continuous and there is no need to satisfy the normal distribution [75]. In a logistic regression analysis, the dependent variable has values of 0 and 1, representing nonlandslide occurrences and landslide occurrences, respectively. The LR model can be expressed as the following equation:

(10) $$P=\frac{\exp(Z)}{1+\exp(Z)},$$

where *P* stands for the probability of landslide occurrences, whose value ranges from 0 to 1; *Z* is calculated by the following equation with the output values range from −∞ to +∞:

(11) $$Z=B_{0}+B_{1}X_{1}+B_{2}X_{2}+\cdots \cdots +B_{n}X_{n},$$

where *n* is the number of independent variables; *B_i_* (*i* = 1, 2, 3, ..., *n*) is the logistic regression coefficient and *X_i_* are the values of the *n* explanatory variables; and *B*_0_ is a constant.

Because the values of *S_ij_* were obtained from the IOE model and the dimension of *S_ij_* is uniform, it can avoid the linear correlation between landslides and explanatory variables and also reduce the noise in modeling. In this study, the 10 explanatory variables were reclassified with the corresponding *S_ij_* values. Then, the values of *S_ij_* were regarded as the input data to build the hybrid model (LR–IOE) through the forward stepwise method to calculate *B*_0_ and *B_i_*.

### 4.4. Integration of Support Vector Machine and Index of Entropy Model

The basic theory of the support vector machine is to transform the input space into high-dimensional space through an inner product function using the training data [76]. The support vectors are defined as the training samples that have the smallest distance from the optimal hyper plane [40]. In this study, SVM is designed to solve binary classification problems, which means that the positive and negative samples exist at the same time.

Consider a set of training vectors *x_i_* (*i* = 1, 2, 3, ..., *n*), and *x_i_* consists of two types denoted as *y_i_* = ±1 [77]. SVM aims to search an *n*-dimensional hyperplane distinguishing the two categories; meanwhile, ensure that these two classes are farthest from the hyperplane. Using mathematical formulas, this can be expressed as follows:

(12) $$P=\frac{1}{2}{\left\|w\right\|}^{2},$$

followed by constraints:

(13) $$y_{i}\left(\left(w\times x_{i}\right)+k\right)\geq 1$$

where ‖w‖ stands for the norm of hyperplane normal; *k* is a constant. By applying the Lagrangian multiplier ($\lambda_{i}$), the cost function can be written as:

(14) $$L=\frac{1}{2}{\left\|w\right\|}^{2}-\sum_{i=1}^{n}\lambda_{i}\left(y_{i}((w\times x_{i})+k)-1\right).$$

In addition, slack variable $\xi_{i}$ is applied to solve the nonseparable problems [76]; thus, Equations (12) and (13) can be modified as:

(15) $$y_{i}\left(\left(w\times x_{i}\right)+k\right)\geq 1-\xi_{i},$$

(16) $$L=\frac{1}{2}{\left\|w\right\|}^{2}-\frac{1}{vn}\sum_{i=1}^{n}\xi_{i},$$

where *v* stands for misclassification, with values ranging from 0 to 1. In addition, by introducing a kernel function, the nonlinear decision boundary can be calculated. In the current research, the following kernel function, namely, the radial basis function (RBF), which is considered to be one of the most powerful kernels [78], is selected to calculate LSI_SVM_ and produce landslide susceptibility map. The radial basis function is shown as follows:

(17) $$K(x_{i},x_{j})=\exp(-\delta {\left\|x_{i}-x_{j}\right\|}^{2}),\delta >0,$$

where δ accounts for the width of the Gaussian kernel function [19].

Similarly, the *S_ij_* was used to be the input data for the SVM model and then build the new hybrid model (SVM–IOE).

### 4.5. The ROC Curve

To test the performance of LSMs obtained by the three models, the receiver operating characteristics (ROC) curve was applied. Based on a series of different dichotomies (cutoffs or decision thresholds), the ROC curve plots 1—specificity as *X*-axis and sensitivity as *Y*-axis, which can be expressed as:

(18) $$X-\mathrm{axis}=1-\mathrm{specificity}=1-\left[\frac{\mathrm{TN}}{\mathrm{TN}+\mathrm{FP}}\right],$$

(19) $$Y-\mathrm{axis}=1-\mathrm{sensitivity}=\frac{\mathrm{TP}}{\mathrm{TP}+\mathrm{FN}},$$

where TP represents true positive, TN is true negative, FP is false positive, and FP is false negative [79]. The quality of these three models predicting the occurrences or non-occurrences of landslide can be measured by the area under the ROC curve (AUC) [9]. The AUC values range from 0 to 1; in addition, if the AUC value is closer to 1, it indicates that the accuracy of model prediction is higher. Conversely, if AUC value is less than 0.5, and closer to 0, it indicates that the model prediction has no practical value [80].

## 5. Results

### 5.1. Assessment of Explanatory Variables

In this study, the training dataset was used to evaluate explanatory variables and the Pearson correlation coefficient between pairs of explanatory variables was calculated (Table 2). It can be seen from the results that the lowest PCC value is −0.009, which happened between altitude and NDVI, and the highest PCC value happened between slope aspect and distance to rivers (0.368). All PCC values are less than 0.7.

The calculation results of VIF and TOL are shown in Table 3. It can be observed that the maximum VIF value is 1.926 and the minimum TOL value is 0.519, which means all the explanatory variables can be applied for landslide susceptibility modeling.

### 5.2. Result of IOE Model

The calculation method of *W_j_* has already been described in Section 4.2, Equations (3)–(8), and the results are shown in Table 4. The *FR_ij_* values shown in Table 4 were used as the input data for slope aspect, lithology, and land use. For the remaining explanatory variables, the original (continuous) data were used as input data to compute the IOE values. Based on the obtained results, the landslide susceptibility index for the IOE model (LSI_IOE_) was calculated using Equation (9) and was written as follows:

LSI_IOE_ = (slope aspect × 0.084) + (slope angle × 0.064) + (altitude × 0.874) + (lithology × 0.119) + (mean annual precipitation × 0.232) + (distance to roads × 0.517) + (distance to rivers × 0.127) + (distance to faults × 0.030) + (land use × 0.974) + (NDVI × 0.303) (20)

**Figure 3:**
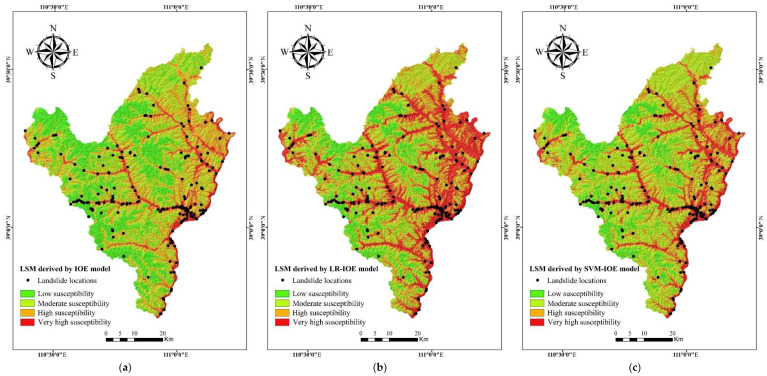
Landslide susceptibility map derived from: (**a**) The IOE model; (**b**) logistic regression (LR)–IOE model; (**c**) support vector machine (SVM)–IOE model.

In the end, all of the 10 explanatory variables were used to build the IOE model, and LSI_IOE_ values range from −10.37 to 11.67. LSI_IOE_ values reflect the probability of landslide occurrence. In other words, the closer the values of LSI_IOE_ are to 11.67, the higher the probability of landslide occurrence, and the values of LSI_IOE_ are close to −10.37, indicating that the probability of occurrence of a landslide is lower. Then, the natural break method was applied to classify the final LSM produced by the IOE model into four categories, which were low (−10.37 to −4.33), moderate (−4.33 to −1.65), high (−1.65 to 1.64), and very high (1.64 to 11.67) (Figure 3a). Additionally, the area percentage of low, moderate, high, and very high regions is 31.24%, 16.39%, 33.23%, and 19.14%, respectively.

### 5.3. Result of LR–IOE Model

The calculation method of *Z* has already been described in Section 4.2, Equations (3)–(8). The *S_ij_* values shown in Table 4 were used as the input data for all 10 explanatory variables through the reclassification method to build the LR–IOE model and to compute *B_0_* and *B_i_* using SPSS software. Based on the results, Equation (11) can be written as follows:

*Z* = 2.345 + (slope aspect × 0.061) + (slope angle × 0.043) + (altitude × −0.252) + (lithology × −0.013) + (mean annual precipitation × 0.239) + (distance to roads × −0.533) + (distance to rivers × −0.269) + (distance to faults × 0.110) + (land use × 0.061) + (NDVI × −0.354) (21)

Subsequently, the LSI_LR__–__IOE_ values were obtained, which range from 0.016 to 0.983. LSI_LR__–__IOE_ values reflect the probability of landslide occurrence. In other words, the closer the values of LSI_LR__–__IOE_ are to 1, the higher the probability of landslide occurrence, and the values of LSI_LR__–__IOE_ are close to 0, indicating that the probability of landslide occurrence is lower. Similarly, the natural break method was applied to classify the final LSM produced by the LR–IOE model into four categories: Low (0.016–0.248), moderate (0.248–0.445), high (0.445–0.688), and very high (0.688–0.983) (Figure 3b). In addition, the area percentage of low, moderate, high, and very high is 16.77%, 33.06%, 21.05%, and 29.12%, respectively.

### 5.4. Result of SVM–IOE Model

In the current research, the parameters of the radial basis function were selected by the grid search method with 10-fold cross validation, and then the entropy was regarded as the input data to calculate the LSI_SVM–IOE_ values based on SVM–IOE model. The LSI_SVM–IOE_ values range from 0.061 to 0.984. The closer the values are to 1, the higher the probability of landslide occurrence, and the values of LSI_SVM–IOE_ are close to 0, indicating that the probability of landslide occurrence is lower. Then, the natural break method was applied to classify the final LSM produced by the SVM–IOE model into four categories: Low (0.061–0.271), moderate (0.271–0.437), high (0.437–0.658), and very high (0.658–0.984) (Figure 3c). The area percentage of low, moderate, high, and very high is 15.08%, 29.56%, 33.39%, and 21.97%, respectively.

### 5.5. Validation of Landslide Susceptibility Maps

In the current study, the ROC curve was used to validate and compare the performance of the IOE, LR–IOE, and SVM–IOE models. The final AUC values represent the success and prediction rate derived from the training and validating dataset, respectively.

**Figure 4:**
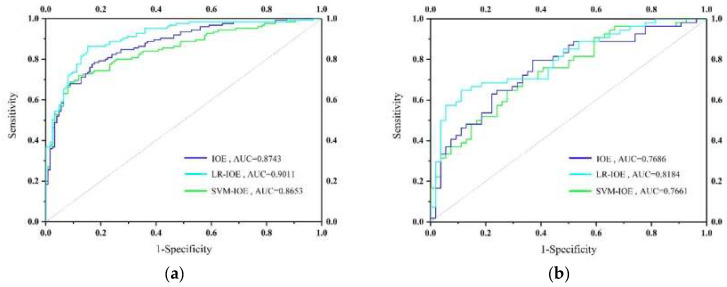
Receiver operating characteristics (ROC) curves of models: (**a**) Training dataset; (**b**) validating dataset.

In the end, for success rate results, the AUC values for the IOE, LR–IOE, and SVM–IOE models were observed to be 0.8743, 0.9011, and 0.8653, respectively (Figure 4a). That is to say, the training accuracy of the susceptibility maps is 87.43%, 90.11%, and 86.53%, respectively. In terms of prediction rate results, the AUC values for the IOE, LR–IOE, and SVM–IOE models were found to be 0.7686, 0.8184, and 0.7661, respectively (Figure 4b). In other words, the prediction accuracy of the susceptibility maps is 76.86%, 81.84%, and 76.61%, respectively.

Generally, the results of both the success rate and prediction rate express reasonable and practical accuracies in the current research. However, the LR–IOE model shows the best result for the current study.

## 6. Discussion

Spatial prediction of landslides is a critical process in the study of landslides and the accuracy of prediction will be affected by the models that we used, and the input data extracted from explanatory variables. However, there is no definitive conclusion about the methods used to select and evaluate explanatory variables. Therefore, it is necessary to investigate the methods which will help us to obtain reasonable conclusions. In this study, we calculated the IOE and PCC to assess 10 explanatory variables, and evaluated three classification models, namely, IOE, LR–IOE, and SVM–IOE, for landslide susceptibility mapping.

According to PCC values (Table 2), all 10 factors are less than 0.7, which means these 10 factors cannot generate noise in landslide susceptibility modeling. From the index of entropy (Table 4), we can see the residential areas have the highest value (7.555), which means that most landslides occurred in this region. We believe that the reason for this condition is the concentration of population and the fact that human engineering activities are intense in this area. Similarly, the closer to the road, the higher the frequency of landslides that occurred was. For the slope aspect, most landslides occurred on south-facing slopes; the reason for this condition may be the climate, and the same results were also reported by the authors of [37] (p. 82). The category C (Siltstone, sandstone, mudstone, shale, coal seam, glutenite) in lithology is the region where the largest number of landslides has occurred. This may be due to the softness of sandstone and siltstone structures and strong weathering erosion. In the case of slope angle and mean annual precipitation, the rate of landslide occurrence is roughly proportional to them. The reason may be that a large amount of water infiltrate increases the water content and weight of the rock and soil mass and increases the sliding force of the rock and soil mass, and the steeper the slope, the stronger the slip force of the rock and soil mass. Interestingly, with the values of distance to faults, distance to rivers, distance to roads, altitude, and NDVI increasing, the IOE is gradually decreasing. The reason for this phenomenon is that road construction usually causes instability, while roads in the study region are generally built at low altitudes and away from faults. The root of the vegetation is conducive to the stability of the soil, while the erosion of the rivers will affect the stability of the slope. These conditions are roughly the same as those observed in the field.

In this study, the selection of explanatory variables was based on previous studies and field observations, which will cause interference from human factors. In addition, although we calculated all the *W_j_* values for the 10 explanatory variables, it is not clear how much the method developed in the work is sensitive to the number of the classes and to the choice of the breaking points. Therefore, this is the focus of future research.

As shown in Figure 4, we can see the AUC value of the LR–IOE model is the highest among the three models, whether it is for the success or prediction rate, which means that the LR–IOE model performs best in landslide susceptibility mapping in this study. However, the AUC value of the SVM–IOE model is the lowest, which may be due to the fact that the SVM–IOE model is more dependent on the selection of the kernel function, and there is no objective way to solve it.

**Figure 5:**
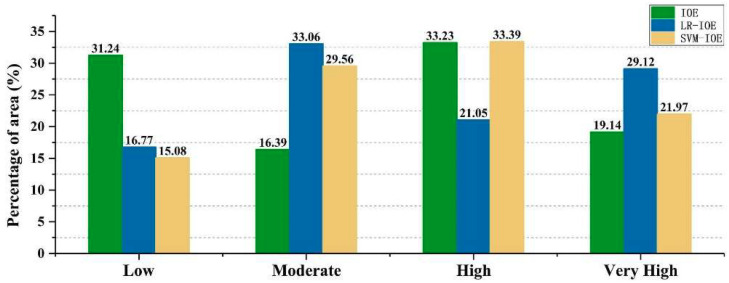
Percentages of different landslide susceptibility classes for the three models.

In terms of the proportion of the final susceptibility mapping results (Figure 5), it can be observed that the proportion of high and very high regions obtained by the three models is about 52%. Among them, the LR–IOE model has the lowest result (50.17%), which implies an efficient result corresponding to the LR–IOE model, and it can also improve the efficiency of decision-making and reduce costs.

## 7. Conclusions

In this present study, the IOE model, LR–IOE model, and SVM–IOE model were used to obtain landslide susceptibility maps for the Fugu County of Shaanxi Province, China. Ten explanatory variables, namely, altitude, slope aspect, mean annual precipitation, slope angle, lithology, distance to roads, land use, distance to rivers, distance to faults, and NDVI, were selected and the potential multicollinearity problem among them was detected by PCC, VIF, and TOL. The results of the analysis showed that there are no potential multicollinearity problems between these 10 factors and they are available for landslide susceptibility modeling. A total of 194 landslides, including landslides recognized from extensive field investigations and historical landslide records, and 194 nonlandslide points were also randomly generated. To build the models, 272 (70%) landslide and nonlandslide points were randomly selected and the remaining 116 (30%) landslide and nonlandslide points were applied for validating purposes. A natural break method was used to split the study region into four categories: Low, moderate, high, and very high. In the end, the performance of the achieved landslide susceptibility maps was evaluated using AUC values.

In terms of the success rate presented by the AUC values, the LR–IOE model has the highest training accuracy (90.11%), followed by the IOE model (87.43%) and the SVM–IOE model (86.53%). As for the prediction rate, the LR–IOE model has the highest training accuracy (81.84%), followed by the IOE model (76.86%) and the SVM–IOE model (76.61%). Thus, the results prove that these three models present good performance in landslide susceptibility mapping. The LR–IOE model performed best for this research and is more suitable for landslide susceptibility mapping in the study area.

The results of this study provide available information for the engineers, decision makers, and urban planners in this study region.

## Figures and Tables

**Table 1:** Lithological units of study area.

Category	Geological Age	Code	Main Lithology
A	Holocene	Q4	Sand, gravel, loess
	Pleistocene	Q3	Loess, gravel
B	Pliocene	N2j	Sandy clay
	Pliocene	N2b	Quartz sand, clay
C	Middle Jurassic	J2y	Siltstone, sandstone, mudstone, shale, coal seam
	Late Jurassic	J1f	Mudstone, glutenite
D	Early Triassic	T3w	Mudstone, shale, coal seam
	Early Triassic	T2-3y	Glutenite, mudstone, shale, siltstone
	Middle Triassic	T2z	Sandstone, mudstone
	Late Triassic	T1h	Medium-fine sandstone, siltstone, mudstone
	Late Triassic	T_1_l	Sandstone, mudstone
E	Early Permian	P_2_s	Glutenite, sandstone, mudstone
	Early Permian	P_2_sh	Mudstone, silty mudstone, sandstone, clay minerals, siliceous
	Late Permian	P_1_sh	Feldspar quartz sandstone, conglomerate, sandstone, mudstone, shale
	Late Permian	P_1_s	Mudstone, shale, sandstone, coal seam
F	Carboniferous	C_2_t	Calcaremaceous sandstone, coal seam, mudstone

**Table 2:** Pearson correlation coefficient between pairs of explanatory variables.

Explanatory Variables	Slope Aspect	Slope Angle	Altitude	Lithology	Mean Annual Precipitation	Distance to Roads	Distance to Rivers	Distance to Faults	Land Use
Slope aspect	1								
Slope angle	0.037	1							
Altitude	0.116	0.003	1						
Lithology	0.165	0.170	0.010	1					
Mean annual precipitation	0.140	0.100	−0.021	0.025	1				
Distance to roads	0.280	0.067	0.079	0.048	0.205	1			
Distance to rivers	0.368	0.104	0.112	−0.010	0.004	0.160	1		
Distance to faults	0.320	0.054	−0.070	0.075	0.024	0.034	0.119	1	
Land use	0.123	−0.116	0.087	0.053	0.287	0.050	0.084	0.019	1
NDVI	0.038	0.011	−0.009	0.179	0.146	−0.065	−0.055	0.047	0.082

**Table 3:** VIF and tolerances for explanatory variables.

Explanatory Variables	VIF	Tolerances
Slope angle	0.657	1.523
Slope aspect	0.962	1.040
Altitude	0.790	1.265
Distance to rivers	0.687	1.455
Distance to roads	0.573	1.746
Distance to faults	0.909	1.100
NDVI	0.770	1.298
Land use	0.910	1.099
Lithology	0.519	1.926
Mean annual precipitation	0.611	1.637

**Table 4:** Spatial relationship between each landslide explanatory variable and landslide by the index of entropy (IOE) model.

Explanatory Variables	Classes	No. of Pixels in Domain	% Percentage of Domain	No. of Landslide	% Percentage of Landslides	*FR_ij_*	*S_ij_*	*M_j_*	*M_jmax_*	*I_j_*	*W_j_*	*B_i_*
Slope aspect	Flat	736	0.021	0	0.000	0.000	0.000	2.870	3.170	0.095	0.084	0.061
	North	436,175	12.234	9	6.569	0.537	0.067					
	Northeast	478,233	13.413	21	15.328	1.143	0.143					
	East	453,979	12.733	9	6.569	0.516	0.065					
	Southeast	435,974	12.228	32	23.358	1.910	0.239					
	South	492,245	13.806	15	10.949	0.793	0.099					
	Southwest	471,646	13.229	25	18.248	1.379	0.173					
	West	413,514	11.598	13	9.489	0.818	0.103					
	Northwest	382,820	10.737	13	9.489	0.884	0.111					
Slope angle (°)	0–6.65	434,598	12.190	16	11.679	0.958	0.135	2.445	2.585	0.054	0.064	0.043
	6.65–11.40	954,012	26.758	31	22.628	0.846	0.119					
	11.40–16.39	937,524	26.296	25	18.248	0.694	0.098					
	16.39–22.09	640,546	17.966	28	20.438	1.138	0.161					
	22.09–29.45	349,550	9.804	14	10.219	1.042	0.147					
	29.45–60.57	249,092	6.987	23	16.788	2.403	0.339					
Altitude (m)	761–903	71,702	2.011	26	18.978	9.437	0.675	1.577	2.807	0.438	0.874	−0.252
	903–984	354,938	9.955	26	18.978	1.906	0.136					
	984–1054	796,328	22.335	27	19.708	0.882	0.063					
	1054–1124	851,004	23.869	26	18.978	0.795	0.057					
	1124–1194	989,546	27.755	28	20.438	0.736	0.053					
	1194–1262	487,438	13.672	4	2.920	0.214	0.015					
	1262–1423	14,366	0.403	0	0.000	0.000	0.000					
Lithology	Category A	80,805	2.266	1	0.730	0.322	0.109	1.963	2.585	0.240	0.119	−0.013
	Category B	650,270	18.239	14	10.219	0.560	0.189					
	Category C	2,029,316	56.918	115	83.942	1.475	0.497					
	Category D	736,194	20.649	6	4.380	0.212	0.072					
	Category E	65,704	1.843	1	0.730	0.396	0.134					
	Category F	3033	0.085	0	0.000	0.000	0.000					
Mean annual precipitation (mm/y)	<360	63,468	1.780	2	1.460	0.820	0.081	2.357	2.807	0.160	0.232	0.239
	360–380	630,456	17.683	5	3.650	0.206	0.020					
	380–400	537,282	15.070	20	14.599	0.969	0.096					
	400–420	850,900	23.866	22	16.058	0.673	0.066					
	420–440	999,895	28.045	44	32.117	1.145	0.113					
	440–460	451,402	12.661	39	28.467	2.248	0.222					
	>460	31,919	0.895	5	3.650	4.077	0.042					
Distance to roads (m)	<200	385,498	10.812	77	56.204	5.198	0.617	1.609	2.322	0.307	0.517	−0.533
	200–400	311,580	8.739	20	14.599	1.670	0.198					
	400–600	282,125	7.913	9	6.569	0.830	0.099					
	600–800	248,289	6.964	4	2.920	0.419	0.050					
	>800	2,337,830	65.571	27	19.708	0.301	0.036					
Distance to rivers (m)	<200	1,108,722	31.097	86	62.774	2.019	0.501	1.956	2.322	0.158	0.127	−0.269
	200–400	881,383	24.721	26	18.978	0.768	0.191					
	400–600	642,145	18.011	12	8.759	0.486	0.121					
	600–800	389,497	10.925	7	5.109	0.468	0.116					
	>800	543,575	15.246	6	4.380	0.287	0.071					
Distance to faults (m)	<2000	526,624	14.771	19	13.869	0.939	0.190	2.251	2.322	0.030	0.030	0.110
	2000–4000	459,271	12.882	10	7.299	0.567	0.115					
	4000–6000	431,651	12.107	14	10.219	0.844	0.171					
	6000–8000	344,339	9.658	20	14.599	1.512	0.307					
	>8000	1,803,437	50.583	74	54.015	1.068	0.217					
Land use	Water	13,266	0.372	0	0.000	0.000	0.000	1.258	2.322	0.458	0.974	0.061
	Residential areas	86,117	2.415	25	18.248	7.555	0.711					
	Bare land	178,0712	49.945	71	51.825	1.038	0.098					
	Forest/Grassland	1,317,845	36.963	17	12.409	0.336	0.032					
	Farmland	367,382	10.304	24	17.518	1.700	0.160					
NDVI	−0.39 to −0.019	278,430	7.809	40	19.197	3.739	0.577	1.779	2.322	0.234	0.303	−0.354
	−0.019 to 0.063	988,700	27.731	38	27.737	1.000	0.154					
	0.063–0.134	1,233,777	34.605	43	31.387	0.907	0.140					
	0.134–0.216	837,512	23.491	12	8.759	0.373	0.058					
	0.216–0.607	226,903	6.364	4	2.920	0.459	0.071					

*B*_0_ is 2.345.
